# 

**Published:** 1881-12

**Authors:** 


					﻿DECEMBER, 1881.
TWENTY-EIGHTH YEAR.
HALL'S
JOURNAL
or
HEALTH
E. IL GIBBS. A.M.. M.D.. Editor.
Nq. 141 EIGHTH STREET (Near Broadway), NEW YORK.
• TERMS : $2 a Year, or $1 for Six Months. Single uumier, 20 cts.
				

